# Supplementary material for: The Impact of Nutritional Therapy in the Management of Overweight/Obese PCOS Patient Candidates for IVF
Source: Nutrients. 2023 Oct 20;15(20):4444. doi: 10.3390/nu15204444 (PMC10609803; doi:10.3390/nu15204444)
Supplement: Supplementary file 1 [file nutrients-15-04444-s001.zip › nutrients-2640527-supplementary.pdf]

Table S1. Approved vegetables (raw or steamed or boiled, baked or grilled).

Unlimited vegetables	Limited vegetables
- Swiss chard	asparagus
- cauliflower	- broccoli
- Chinese cabbage	- Roman broccoli
- green cabbage	- fried artichokes
- red and green radicchio	- cardoons
- fennel	- Brussels sprout
- mushrooms	- green beans
- Belgian lettuce	- eggplant
- lettuce	- yellow peppers
- radish	- red peppers
- escarole	- green peppers
- spinach	- tomatoes
- celery	- turnips
- raw artichokes	- dandelion greens
- soybean sprouts	- yellow squash
- coppery	
- cucumber	
- squash blossoms	
- cabbage	
- endive	
- turnip greens	
- chicory	
- valerian	
- zucchini	
- arugul	

Table S2. Contents of supplements.

Contents of supplements	Maximum dose/day
Potassium	2000 mg
Calcium	800 mg
Magnesium	375 mg
Vitamin C	120 mg
Zinc	10 mg
Vitamin E	12 mg
Coenzyme Q10	20 mg
Niacin	16 mg
Manganese	2 mg
Selenium	55 μg
Vitamin A	800 μg
Pantothenic acid	6 mg
Vitamin B6	4 mg
Vitamin D	10 μg
Cupper	1 mg
Vitamin B12	2.5 μg
Riboflavin	1.4 mg
Thiamine	1.1 mg
Folic acid	400 μg
Chromium	40 μg
Biotin	50 μg

Table S3. Typical daily patterns of the VLCKD intensive phase, VLCKD transition phase and Mediterranean diet.

	VLCKD intensive phase	VLCKD transition phase	Mediterranean diet
**Breakfast**	substitute food A + coffee/tea no sugar	substitute food A + coffee/tea with no sugar	20 g semi-skimmed cow's milk + coffee/tea with no sugar + 50 g whole-wheat rusks/50 g whole-wheat cookies
**Snack**	-	-	300 g of fresh fruit **or** 160 g of low-fat yogurt
**Lunch**	substitute food B + 30 g of accompanying food + vegetables **or** substitute food C + vegetables	substitute food B + vegetables **or** 120 g meat/150 g fish+ vegetables	60 g bread/70 g whole-wheat bread/50 g brown rice/50 g whole-wheat pasta + 200 g fresh vegetables **or** 100 g low-fat cheese/1 egg or 40 g ham (once a week)+ 15 g EVO
**Snack**	substitute food D	substitute food D **or** 30 g dried fruits	300 g fresh fruit
**Dinner**	substitute food C + vegetables **or** substitute food B + 30 g accompanying food + vegetables	120 g meat/150 g fish+ vegetables **or** substitute food B + vegetables	300 g potatoes/80 g bread/100 g whole grain + 50 g lean meat/60 g fish + 200 g fresh vegetables + 15 g EVO

Table S4. Nutritional information panel of substitute food A.

Nutritional values per serving (40 g)	Substitute food A
Energy	671 kJ/ 167 kcal
Fats	9.1 g
of which saturated fatty acids	1.7 g
Carbohydrates	7.1 g
of which sugars	0.1 g
of which polyols	5.8 g
Fibers	4.0 g
Proteins	14 g
Salt	0.04 g

Table S5. Nutritional information panel of substitute food B.

Nutritional values per serving (50 g)	Substitute food B
Energy	622 kJ/ 149 kcal
Fats	1.5 g
of which saturated fatty acids	0.5 g
Carbohydrates	4.9 g
of which sugars	0 g
Fibers	17 g
Proteins	20 g
Salt	0.01 g

Table S6. Nutritional information panel of substitute food C.

Nutritional values per serving (30.5 g)	Substitute food C
Energy	464 kJ/ 110 kcal
Fats	1.7 g
of which saturated fatty acids	0.8 g
Carbohydrates	5.2 g
of which sugars	0.5 g
of which starches	4.7 g
Fibers	0.8 g
Proteins	18 g
Salt	2.2 g

Table S7. Nutritional information panel of substitute food D.

Nutritional values per serving (30 g)	Substitute food D
Energy	490 kJ/ 117 kcal
Fats	3.7 g
of which saturated fatty acids	0.4 g
Carbohydrates	5.0 g
of which sugars	0.7 g
Fibers	4.9 g
Proteins	14 g
Salt	0.87 g
